# 3,3′Diindolylmethane Suppresses Vascular Smooth Muscle Cell Phenotypic Modulation and Inhibits Neointima Formation after Carotid Injury

**DOI:** 10.1371/journal.pone.0034957

**Published:** 2012-04-10

**Authors:** Hongjing Guan, Lihua Zhu, Mingyue Fu, Da Yang, Song Tian, Yuanyuan Guo, Changping Cui, Lang Wang, Hong Jiang

**Affiliations:** 1 Department of Cardiology, Renmin Hospital of Wuhan University, Wuhan, People's Republic of China; 2 Cardiovascular Research Institute of Wuhan University, Wuhan, People's Republic of China; Inserm, France

## Abstract

**Background:**

3, 3′diindolylmethane (DIM), a natural phytochemical, has shown inhibitory effects on the growth and migration of a variety of cancer cells; however, whether DIM has similar effects on vascular smooth muscle cells (VSMCs) remains unknown. The purpose of this study was to assess the effects of DIM on the proliferation and migration of cultured VSMCs and neointima formation in a carotid injury model, as well as the related cell signaling mechanisms.

**Methodology/Principal Findings:**

DIM dose-dependently inhibited the platelet-derived growth factor (PDGF)-BB-induced proliferation of VSMCs without cell cytotoxicity. This inhibition was caused by a G0/G1 phase cell cycle arrest demonstrated by fluorescence-activated cell-sorting analysis. We also showed that DIM-induced growth inhibition was associated with the inhibition of the expression of cyclin D1 and cyclin-dependent kinase (CDK) 4/6 as well as an increase in p27^Kip1^ levels in PDGF-stimulated VSMCs. Moreover, DIM was also found to modulate migration of VSMCs and smooth muscle-specific contractile marker expression. Mechanistically, DIM negatively modulated PDGF-BB-induced phosphorylation of PDGF-recptorβ (PDGF-Rβ) and the activities of downstream signaling molecules including Akt/glycogen synthase kinase(GSK)3β, extracellular signal-regulated kinase1/2 (ERK1/2), and signal transducers and activators of transcription 3 (STAT3). Our in vivo studies using a mouse carotid arterial injury model revealed that treatment with 150 mg/kg DIM resulted in significant reduction of the neointima/media ratio and proliferating cell nuclear antigen (PCNA)-positive cells, without affecting apoptosis of vascular cells and reendothelialization. Infiltration of inflammatory cells was also inhibited by DIM administration.

**Conclusion:**

These results demonstrate that DIM can suppress the phenotypic modulation of VSMCs and neointima hyperplasia after vascular injury. These beneficial effects on VSMCs were at least partly mediated by the inhibition of PDGF-Rβ and the activities of downstream signaling pathways. The results suggest that DIM has the potential to be a candidate for the prevention of restenosis.

## Introduction

Atherosclerosis is the primary pathological event leading to decreases in arterial lumen size. The thrombotic complications of atherosclerosis, such as myocardial infarction and stroke, are the leading causes of death in both middle- and high-income countries and are among the top five causes of death in low-income countries [1]. Great efforts have been made to find efficient therapies to overcome atherosclerotic obstructive disease. Percutaneous coronary intervention (PCI), which has advanced over the past decades, can restore blood flow in these vessels. Nevertheless, restenosis of the artery following PCI is the major factor hampering the long-term success of the procedure. Drug eluting stents (DES) can reduce the restenosis rate to less than 10% [2], [3]; however, emerging evidence suggests that DES has the potential drawback of impairing reendothelialization and increasing the risk of late thrombosis [4], [5]. These drawbacks have prompted the search for new compounds that can efficiently inhibit restenosis with fewer side effects.

Neointima formation is a crucial process in restenosis. During neointima development after vascular injury, growth and prothrombotic factors released from platelets and leucocytes trigger the migration of vascular smooth muscle cells (VSMCs) from the media to the intima, where they proliferate and undergo phenotypic changes. Excessive VSMC proliferation, migration and phenotypic modulation underlie the major pathophysiologic mechanism responsible for the failure of restenosis after PCI [6], [7]. Therefore, inhibiting VSMC proliferation, migration and phenotypic modulation may provide useful approaches to improve existing therapeutic strategies for restenosis.

Epidemiological studies have shown that increased consumption of vegetables and fruits is associated with a lower risk of all-cause, cancer, and cardiovascular disease death [8], [9]. Phytochemicals harvested from vegetables and fruits have received increasing attention recently, and the use of phytochemicals in combination therapies has been considered as one of several novel treatment approaches. One of the most promising bioactive phytochemicals is indole-3-carbinol (I3C), which is produced from cruciferous vegetables, such as cauliflower and broccoli. In the acidic environment of the stomach, I3C is susceptible to oligomerization and converted into a number of condensation products, including a dimeric product, 3,3′diindolylmethane (DIM), its major active metabolite [10]. DIM has shown inhibitory effects on the growth of a variety of cancer cells, including breast, prostate, thyroid, lung, and cervical cancers, with negligible levels of toxicity [11]–[15].

The molecular mechanism by which DIM confers its biological effects has been extensively investigated. It is becoming clear that DIM has pleiotropic effects on multiple signaling targets related to control of the cell cycle, apoptosis, signal transduction, oncogenesis, hormonal homeostasis, and transcription regulation. In vitro studies have indicated that DIM is a potent inhibitor of complexes of cyclin and cyclin-dependent kinases (CDKs) and is responsible for upregulation of CDK inhibitors. DIM also participates in the regulation of Akt signal transduction. Inhibition of the activation of Akt and its downstream effector, NF-κB, has been reported in prostate cancer cells [11]. Moreover, DIM has been shown to regulate Akt/FOXO3a/androgen receptor signaling, resulting in the alteration of p27^Kip1^ expression [16]. In addition to these antiproliferative effects, DIM inhibits angiogenesis and invasion of tumor cells by repressing the expression of matrix metalloproteinase, adhesion molecules and urokinase-type plasminogen activator [13], [17].

However, whether DIM has a direct effect on VSMC proliferation and migration, in addition to its anticancer properties, remains unknown. In addition, the suitability of DIM for preventing highly proliferative vascular responses, such as postangioplasty restenosis, needs further investigation. In the present study, we demonstrate that DIM causes insufficient regulation of the cell cycle proteins that control the G0/G1/S phase progression of VSMCs and inhibit VSMC proliferation. Moreover, DIM also inhibits the PDGF-induced migration and phenotypic modulation of VSMCs, two other important processes in neointima development. The mechanism by which DIM confers its beneficial effects on VSMCs may be the blockade of PDGF signal cascades. Finally, we demonstrate that oral administration of DIM prevents VSMC proliferation and neointima formation in a mouse model of vascular injury.

## Materials and Methods

### Materials

DIM was purchased from Sigma-Aldrich (St. Louis, MO). Recombinant human platelet-derived growth factor-BB (PDGF-BB) came from Prospec (Rehovot, Israel). The antibodies used to recognize the total levels and phosphorylation of extracellular signal-regulated kinase 1/2 (ERK1/2), Akt, glycogen synthase kinase-3β (GSK-3β) and signal transducer and activator of transcription 3 (STAT3) were obtained from Cell Signaling technology (Danvers, MA). Antibody against total levels and phosphorylation of PDGF-receptor β (PDGF-Rβ), smooth muscle alpha-actin (SM-α-actin), SM22α, CD31were purchased from Abcam (Cambridge, MA). Anti-desmin came from Santa Cruz Biotechnology (Santa Cruz, CA). Antibodies against CDk4, CDk6, cyclin D1, p27^Kip1^, glyceraldehyde-3-phosphate dehydrogenase (GAPDH), and proliferating cell nuclear antigen (PCNA) were purchased from Cell Signaling technology. Antibody against CD45 was purchased from BD Biosceinces (San Diego, CA). Complete protease inhibitor, PhosSTOP, Cell Proliferation Reagent WST-1 and Cell Proliferation ELISA, BrdU (colorimetric) kits were obtained from Roche Diagnostics (Mannheim, Germany). All other reagents were purchased from Sigma-Aldrich, except where specified. For the in vitro study, DIM was dissolved in dimethyl sulfoxide (DMSO). DMSO alone, without DIM, served as a control. DMSO did not show any effect on cell viability, cell proliferation, or related molecular mechanisms (data not shown).

### Cell culture

Primary vascular smooth muscle cells were enzymatically isolated from the thoracic aortas of male Sprague-Dawley rats (100 to 150 g) and grown in DMEM/F12 medium containing 10% fetal bovine serum as previously described [18]. The purity of the VSMCs was determined by the morphological characteristics and positive staining of SM α-actin, the positive cells are over 95%. The cells used in this study were between passages 5 and 12. The VSMCs were grown to 60% and 80% confluence and serum starved for 24 h. Quiescent cells were pretreated with various concentrations of DIM for 1 h prior to stimulation with PDGF-BB (20 ng/ml). Human Umbilical Vein Endothelial Cells (HUVECs) were purchased from ScienCell, the cells were grown in endothelial cell medium (ScienCell). The cells used in this study were between passages 3 and 5.

### Measurement of cell proliferation and DNA synthesis

Cell proliferation was determined by the nonradioactive colorimetric WST-1 assay according to the manufacturer's instructions. The VSMCs (5×10^3^/well) were grown to 60% confluence and growth-arrested in a 96-well microplate. After preincubation with various concentrations of DIM for 1 h, the cells were treated with PDGF-BB (20 ng/ml) in the presence/absence of DIM for 48 h and loaded with WST-1 for the final 2 h. The cell's color intensity was determined at 450 nm. DNA synthesis in VSMCs was assessed by measuring the incorporation of BrdU. The cells were seeded and treated as in the WST-1 assay. BrdU was added for the last 2 h of treatment. BrdU incorporation was measured with a cell proliferation ELISA kit.

### Evaluation of cell viability

Trypan blue exclusion was used to determine the viability of VSMCs and HUVECs. After addition of different concentrations of DIM for 24 h or 48 h, VSMCs or HUVECs were trypsinized and incubated with 0.4% trypan blue dye. Viability was assessed by automated determination of the percentage of cells that are able to exclude trypan Blue with a Countess® Automated Cell Counter (Invitrogen)

### Assays of cell cycle progression

Cell cycle progression was measured using propidium iodide staining with a fluorescence-activated cell-sorting (FACS) analysis. Briefly, cells at 70% confluence were synchronized for 24 h and preincubated with DIM (25 µM) for 1 h, then subsequently treated with PDGF-BB (20 ng/ml) for 24 h. The cells were then trypsinized and fixed with 70% ethanol overnight. The fixed cells were collected by centrifugation, washed once in PBS and incubated with 1 ml propidium iodide (PI) staining buffer (20 µg/ml PI and 50 µg/ml RNase A), and then analyzed with FACS. The cell cycle distributions were analyzed with Multicycle AV software (Phoenix Flow Systems, San Diego, CA).

### Migration assay

The migration assay was performed using the Transwell system (a 6.5-mm polycarbonate membrane with 8-µm pores; Corning, NY). Fifty microliters (5×10^4^) of cells were seeded on the upper chamber and attached for 30 min. The monolayers were then treated for 1 h by adding 50 µl of 2-fold-concentrated DIM solution to the upper chamber and 600 µl of the DIM solution (1×) to the lower chamber. PDGF-BB was added to the bottom chamber as the chemoattractant. The cells were allowed to migrate through the membrane to the lower surface for 6 h. Cells on the upper surface of the membrane that had not migrated were scraped off with cotton swabs, and cells that had migrated to the lower surface were fixed and stained with 0.1% crystal violet/20% methanol and counted. Migrated cell numbers were calculated as the number of migrated cells per high-power field (200×).

### Western blotting

The VSMCs were cultured in a 6-cm diameter dish and grown to 70% to 80% confluence, then starved in serum-free medium for 24 h. The cells were then treated with DIM (25 µM) for 2 h before exposure to 20 ng/ml PDGF-BB for the indicated time. The cells were lysed in RIPA buffer with protease and phosphatase cocktails. Twenty micrograms of protein extract was used for SDS-PAGE. The proteins were then transferred to an Immobilon-FL transfer membrane (Millipore) and probed with various antibodies. After incubation with a secondary IRDye® 800CW-conjugated antibody, the signals were visualized with an Odyssey Imaging System. Specific protein expression levels were normalized to GAPDH for total protein or to total proteins for phosphorylated protein.

### Endovascular carotid artery guidewire injury

All animal experimentation protocols were performed under institutional animal welfare guidelines and approved by the Animal Care and Use Committee of Renmin Hospital of Wuhan University (Permit Number: 00015816). All surgery was performed under sodium pentobarbital anesthesia, and all efforts were made to minimize suffering. Ten-week-old male c57BL/6 mice underwent guidewire endothelial denudation injuries by the insertion of a guidewire (0.38 mm in diameter, No. C-SF-15-15, Cook, Bloomington, Indiana), as previously described [19]. Briefly, with the use of aseptic techniques, the left carotid artery was exposed, and the distal bifurcation of the carotid artery was encircled proximally and ligated distally with 8-0 silk sutures. A guidewire was introduced into the arterial lumen through a transverse arteriotomy made between the sutures. The guidewire was then advanced toward the aortic arch and withdrawn 5 times. After removal of the guidewire, the proximal 8-0 suture was ligated, and the incision was closed. Sham surgery without injury was performed on the right side. After surgery, the mice were fed either a normal rodent chow diet alone or a normal chow diet containing 0.09% DIM (w/w). This diet corresponded to 150 mg/kg DIM if a 30 g mouse consumed 5 g of chow per day. The mice were maintained on these diets for 28 days before they were euthanized. The right and left carotid arteries were isolated, fixed in buffered formalin phosphate and processed for morphometric and immunohistochemical analyses.

### Evaluation of neointima formation

For morphometric analysis, 5-µm-thick paraffin-embedded sections were cut from equally spaced intervals in the middle of injured and control common carotid artery segments and stained with hematoxylin and eosin to demarcate cell types. Fifteen sections from each carotid artery were reviewed and scored under blind conditions. The intimal (I) and medial (M) areas were measured using the Image Pro Plus6.0 program, and the I/M ratios were calculated.

### Immunohistochemistry

Immunostaining for PCNA was performed as previously described [20]. An anti-PCNA monoclonal antibody, complemented by a biotinylated anti-mouse secondary antibody, was applied to perfusion-fixed, paraffin-embedded tissues. The slides were treated with an avidin-biotin block, exposed to DAB with hematoxylin, and analyzed under a light microscope. The data was presented as the number of PCNA-positive-stained cells within the neointima. For fluorescent immunohistochemistry, sections were incubated with primary antibodies at 4°C overnight. After incubation with FITC-conjugated secondary antibody, the slides were observed by fluorescent microscopy. The apoptotic VSMCs were detected by terminal deoxynucleotidyl transferase-mediated dUTP nick endlabelling (TUNEL) according to the supplier's instructions (In situ cell death detection kit, Roche, Mannheim, Germany). Picrosirius red was stained for collagen deposition.

### Statistical analysis

The results were expressed as means±SEM. Statistical analysis was performed using one-way analysis of variance (ANOVA), followed by Dunnett's multiple comparison tests. A value of *P*<0.05 was considered significant.

## Results

### DIM inhibits VSMC proliferation stimulated by PDGF-BB

**Figure 1:**
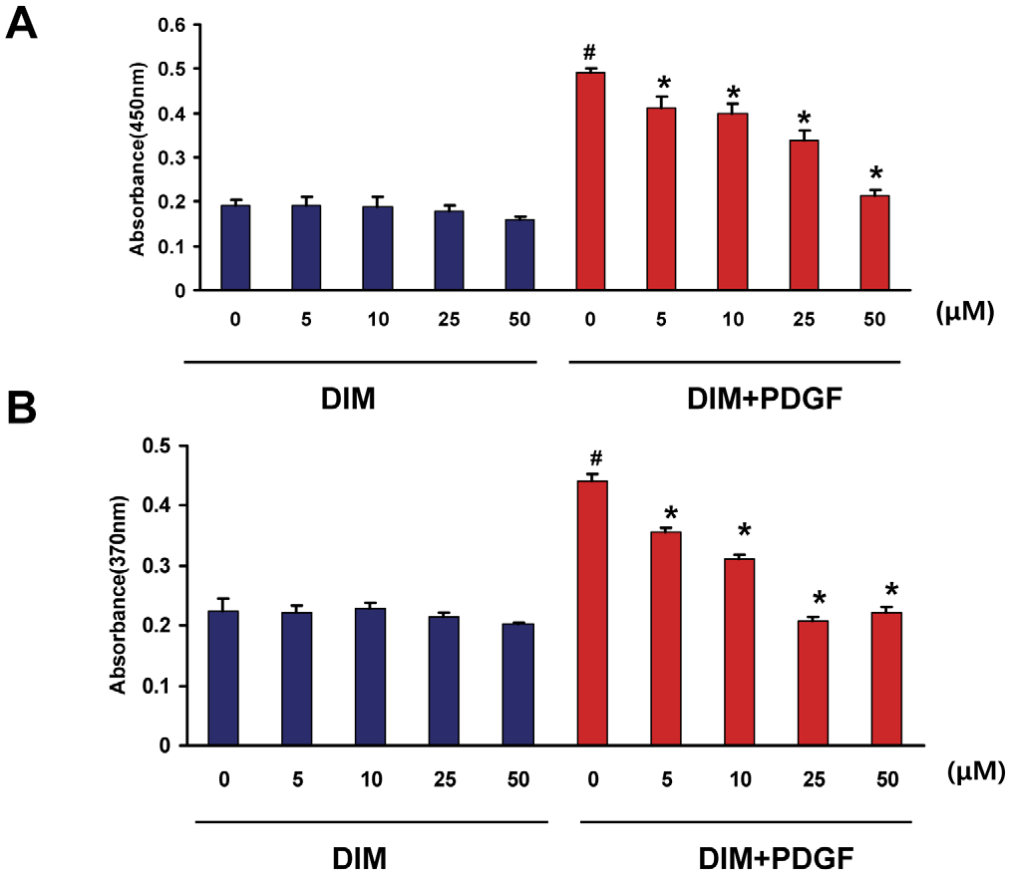
DIM prevents VSMC proliferation and DNA synthesis induced by PDGF-BB. VSMCs were serum starved for 24 h and then treated with the indicated concentrations of DIM (5 to 50 µM) for 48 h in the absence or presence of PDGF-BB (20 ng/ml). A. Cell viability was examined with the WST-1 test. The data are expressed as the mean OD450±SEM (#*P*<0.01 versus control group; *P<0.01 versus PDGF alone; n = 6). B. BrdU incorporation was determined with an ELISA-based assay. DNA synthesis is expressed as the mean OD370±SEM (#*P*<0.01 versus control group; *P<0.01 versus PDGF alone; n = 6).

Abnormal proliferation of VSMCs is known to contribute to vascular lesion formation. DIM has been proven to be an antitumor compound that leads to growth suppression in cancer cells; however, until now it has not been clear whether DIM has a growth-suppressing effect on VSMCs. We first evaluated the effect of DIM on cell proliferation with a WST-1 cell proliferation assay. Stimulation with PDGF-BB for 48 h increased VSMC proliferation by approximately 2.5-fold compared with untreated controls (Figure 1A). DIM prevented the increase in VSMC numbers in a dose-dependent manner. Higher concentrations of DIM (50 µM) almost completely blocked the PDGF-BB-induced cell proliferation. Cells treated with DIM (5 to 50 µM) for 48 h in the absence of PDGF-BB showed no significant difference in the viability of VSMCs compared with the untreated cells, which suggests that DIM is not cytotoxic at the concentrations tested (Figure 1A). We further investigated the inhibitory effect of DIM on DNA synthesis. Figure 1B illustrates the results from a BrdU incorporation assay and shows that PDGF treatment increased DNA synthesis in VSMCs, DIM significantly inhibited DNA synthesis in a dose-dependent manner, and DNA replication was almost completely blocked in VSMCs treated with DIM at concentrations of 25 or 50 µM, compared with PDGF alone (Figure 1B).

### Effect of DIM on viability of VSMC and HUVEC

**Figure 2:**
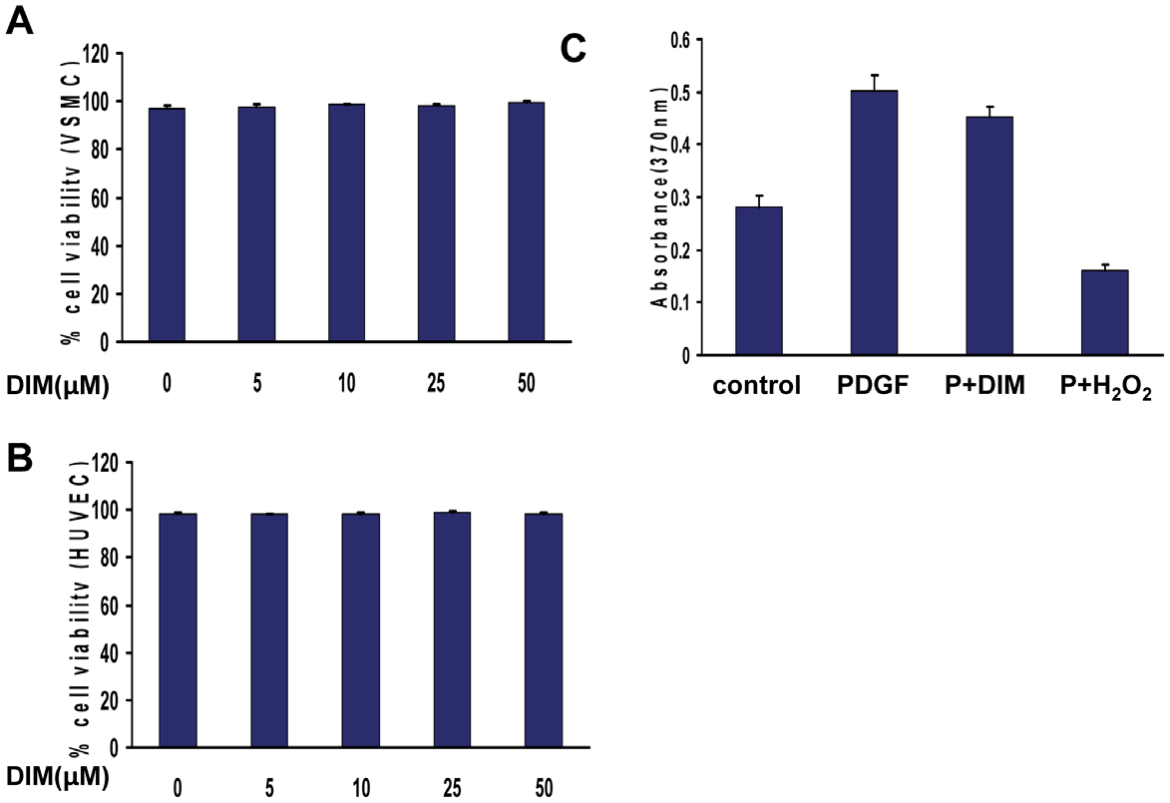
Effect of DIM on viability of VSMC and HUVEC. A. VSMCs were incubated in growth medium in the absence or presence of different concentrations of DIM for 24 hours, and cell viability was evaluated by counting the number of cells that excluded the trypan blue dye (*P* = NS versus control group; n = 4). B, HUVECs were incubated in growth medium in the absence or presence of different concentrations of DIM for 24 hours, and cell viability was evaluated by trypan blue exclusion (*P* = NS versus control group; n = 4). C, VSMCs were incubated in the absence or presence of DIM (25 µM) or H_2_0_2_ (400 µM, serving as positive control) for 24 hours. After 3 washing steps, cells were then stimulated with PDGF-BB for 24 hours, and VSMC proliferation was quantified by BrdU incorporation (*P* = NS versus pretreated with control buffer; n = 6).

To exclude a cell cytotoxicity effect of DIM, we determined cell necrosis by trypan blue exclusion in the absence or presence of DIM. As shown in Figure 2A and Figure S1A, DIM did not induce cell necrosis of VSMCs up to 48 h. Furthermore, the antiproliferative effect of DIM was reversible: cells were serum-starved and synchronized in the presence or absence of DIM for 24 h. After removing DIM, cells were than stimulated with PDGF-BB for 24 h, and BrdU incorporation was quantified. Cells pretreated with DIM (25 µM) showed an almost similar proliferation rate as cells pretreated with control buffer only (Figure 2C). In contrast, cells pretreated with H_2_0_2_ (400 µM) did not proliferate in the presence of PDGF-BB. These data indicate that there is no toxic effect of DIM in the tested concentrations.

Because reendothelialization is a vital process for arterial injury repair, we tested the influence of DIM on endothelial cell viability. HUVECs were incubated in the absence or presence of DIM for 24 h or 48 h before their viability was analyzed according to their ability to exclude trypan Blue (Figure 2B and Figure S1B). The results showed that DIM had no negative influence on endothelial cell viability.

### DIM Prevents Cell Cycle Entry/Progression in the G0/G1 phase

**Figure 3:**
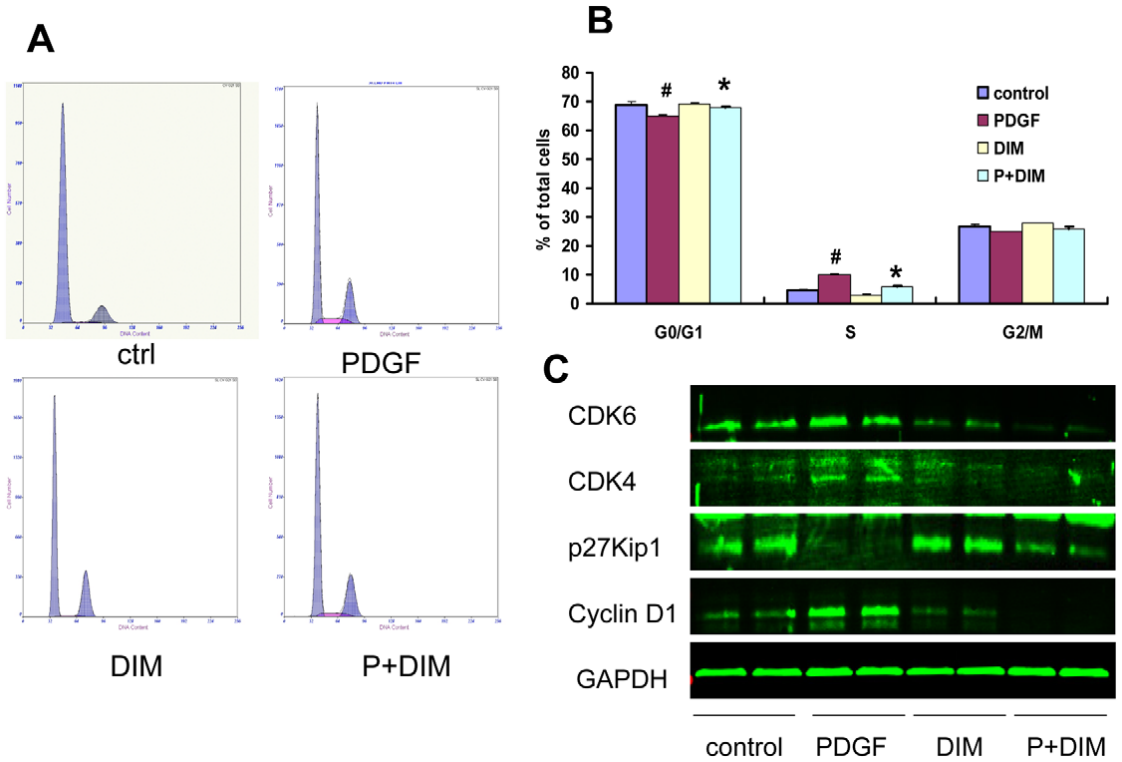
DIM prevents cell cycle progression in VSMCs. VSMCs were grown with DIM (25 µM) in the absence or presence of PDGF-BB (20 ng/ml) for 24 h, and cell cycle distribution was evaluated with flow cytometric analysis. A. Representative cell cycle profiles are shown. B. Quantification of VSMCs in the G0/G1, S, and G2/M phases, as determined by flow cytometric evaluation, is shown (#*P*<0.01 versus control group; *P<0.01 versus PDGF alone; n = 3). C. Cell cycle protein expression was measured with western blot analysis. GAPDH detection served as a loading control.

DIM's effect on cell cycle progression was analyzed to elucidate the mechanisms responsible for its antiproliferative effect. As revealed by flow cytometry, 25 µM DIM significantly increased the fraction of G0/G1 phase cells but decreased the numbers of S phase cells in VSMCs, which indicated that DIM prevented cell cycle entry/progression in the G0/G1 phase (Figures 3A and 3B). This result suggests that DIM acts at the early stages of cell cycle progression.

### DIM inhibits cyclin, CDK expression and p27^Kip1^ degradation induced by PDGF-BB

Cell cycle progression is tightly regulated through specific CDK-cyclin complexes [21]. We found that DIM inhibited cell proliferation and caused G1 cell cycle arrest; cyclin D1, CDK4 and CDK6 may be the targets of DIM action. As shown in Figure 3C, the expression of cyclin D1, CDK4 and CDK6 was induced by PDGF-BB (20 ng/ml), while DIM treatment (25 µM) significantly decreased expression of these molecules (Figure 3C).

We next assessed the effect of DIM on the induction of p27^Kip1^, which inactivates the cyclin-CDK complexes in the G1 phase, leading to cell cycle arrest. P27^Kip1^ was constitutively expressed in serum-starved quiescent VSMCs and was downregulated by PDGF-BB. In contrast, pretreatment with DIM partly restored p27^Kip1^ expression (Figure 3C).

### DIM prevents PDGF-BB-stimulated VSMCs migration

**Figure 4:**
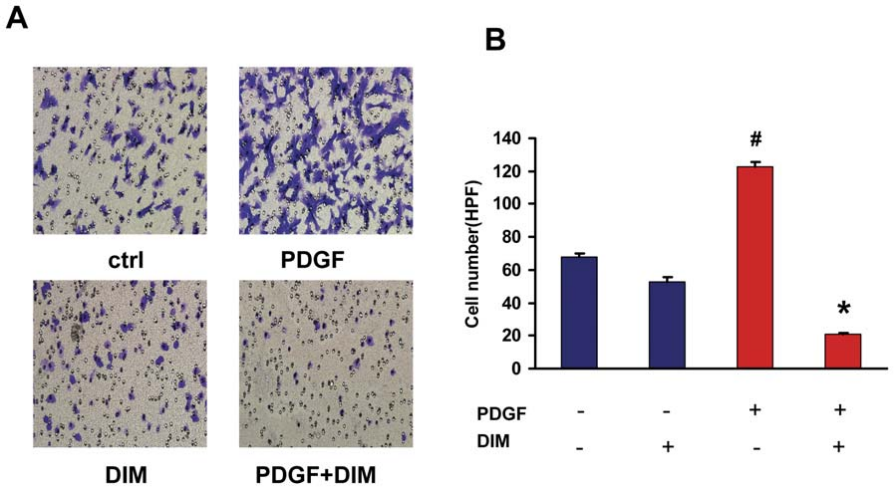
DIM inhibits PDGF-BB-induced cell migration. A. VSMCs were cultured in a cell migration filter insert and stimulated with PDGF-BB for 6 h with or without DIM treatment (25 µM). B. Cellular migration was determined by counting the cells that migrated through the pores. The results are expressed as means±SEM (#*P*<0.01 versus control group; *P<0.01 versus PDGF alone).

Migration of smooth muscle cells from the media to the intimal region is another important component of vascular lesion formation. We next examined whether DIM played a role in regulating VSMCs migration. As indicated in Figure 4A, treatment with PDGF-BB (20 ng/ml) caused an almost 2-fold increase in the migration of VSMCs; however, pretreatment with DIM (25 µM) significantly reduced PDGF-BB-induced migration. The number of migrated cells was significantly decreased by DIM (Figure 4B). These results suggest that DIM is a potent inhibitor of VSMC migration.

### The effect of DIM on phenotypic modulation of VSMCs

**Figure 5:**
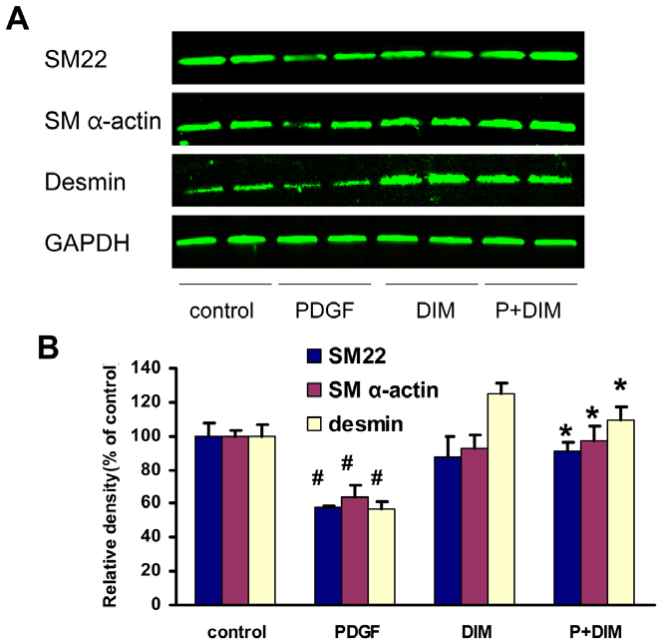
Effect of DIM on the regulation of smooth muscle gene expression. A. VSMCs preincubated for 2 h with DIM and then stimulated with PDGF-BB (20 ng/ml) for 48 h. The protein levels of SM α-actin, SM22α, and desmin were determined with western blot analysis and quantified by densitometry. B. Bar graphs showing the quantification of western blots. The results are expressed as a percentage of control (#*P*<0.05 versus control group; *P<0.05 versus PDGF alone; n = 3).

Pathological conditions such as atherosclerosis, restenosis, and hypertension are associated with phenotypic modulation of VSMCs, which is characterized by loss of contractility, abnormal proliferation, migration, and matrix secretion [22]. Phenotypically modulated VSMCs exhibit decreased expression of a variety of contractile genes, including SM α-actin, SM22α, and desmin. To further evaluate the phenotypic modulation of VSMCs by DIM, western blot analysis was used to detect differentiated phenotype markers. After pretreatment with DIM (25 µM) for 2 h, quiescent VSMCs were stimulated with PDGF-BB (20 ng/ml) for 48 h in the presence/absence of DIM. As indicated in Figure 5, PDGF reduced the protein levels of SM α-actin, SM22α, and desmin. Moreover, pretreatment with DIM partially blocked the repressive effects of PDGF-BB, which suggests that DIM contributes to maintaining the quiescent (differentiated) state of VSMCs.

### Molecular mechanisms involved in DIM inhibition of VSMC proliferation and migration

**Figure 6:**
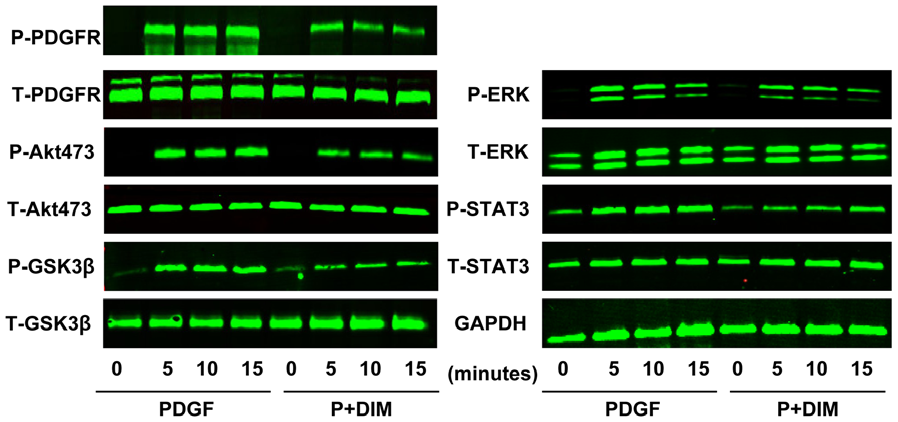
Inhibitory effects of DIM on PDGF-Rβ, Akt, GSK-3β, ERK1/2, and STAT3 activation in the PDGF-BB-stimulated VSMCs. Serum-starved VSMCs were stimulated with PDGF-BB for an indicated time in the absence or presence of DIM (25 µM). The protein levels of phospho-PDGF-Rβ, PDGF-Rβ, phospho-Akt, Akt, phospho-GSK-3β, GSK-3β, phospho-ERK1/2, ERK1/2, phospho-STAT3, and STAT3 were determined with western blot analysis. One representative image out of 3 independently performed experiments is shown.

We then analyzed the molecular mechanisms of the phenotypic modulation of DIM in VSMCs. Previous studies have demonstrated that the mitogen-activated protein kinase (MAPK) and phosphatidylinositol 3-kinase (PI3K)/Akt signal transduction pathways are critically involved in VSMC proliferation and migration. Therefore, we first evaluated the effect of DIM on the PDGF-induced activation of the Akt/GSK3β pathways. The cells were treated with PDGF-BB, and phosphorylation of Akt/GSK3β was detected. Our data showed that PDGF-BB induced rapid and sustained phosphorylation of Akt and GSK3β without affecting their total levels (Figure 6 and Figure S2). We then examined the effects of DIM on the kinetics of PDGF-BB-induced Akt/GSK3β activation. The cells were pretreated with 25 µM DIM for 2 h and then treated with PDGF-BB for the indicated time. As shown in Figure 6, PDGF-BB-induced Akt and GSK3β phosphorylation was slightly but statistically significantly impaired by DIM. The observed inhibitory effects of DIM on PDGF-BB-induced Akt/GSK3β activation were not due to decreases in total protein levels. We then examined the effects of DIM on the PDGF-BB-induced activation of the ERK1/2 pathways. DIM showed a marked decrease in the PDGF-BB induced phosphorylation of ERK1/2 in the same pattern as decrease in Akt phosphorylation (Figure 6 and Figure S2).

STAT3 is thought to play a role in the regulation of cell growth and differentiation. Accordingly, VSMC stimulation with PDGF-BB produced a strong STAT3 activation, as demonstrated by the phosphorylation of STAT3. In contrast, preincubation with DIM (25 µM) significantly attenuated STAT3 phosphorylation (Figure 6 and Figure S2).

DIM was shown to inhibit the downstream components of PDGF-BB such as ERK1/2, Akt and STAT3 phosphorylation with a similar pattern. These results indicated that PDGF-Rβ may be a potential target for DIM. As shown in Figure 6 and Figure S2, pre-treatment with DIM significantly inhibited the PDGF-Rβ phosphorylation(Tyr857) that was induced by PDGF-BB. The observed inhibitory effects of DIM on PDGF-induced PDGF-Rβ phosphorylation were not due to the reduction in total protein levels.

### The effects of DIM on neointima formation and cell proliferation in vivo

**Figure 7:**
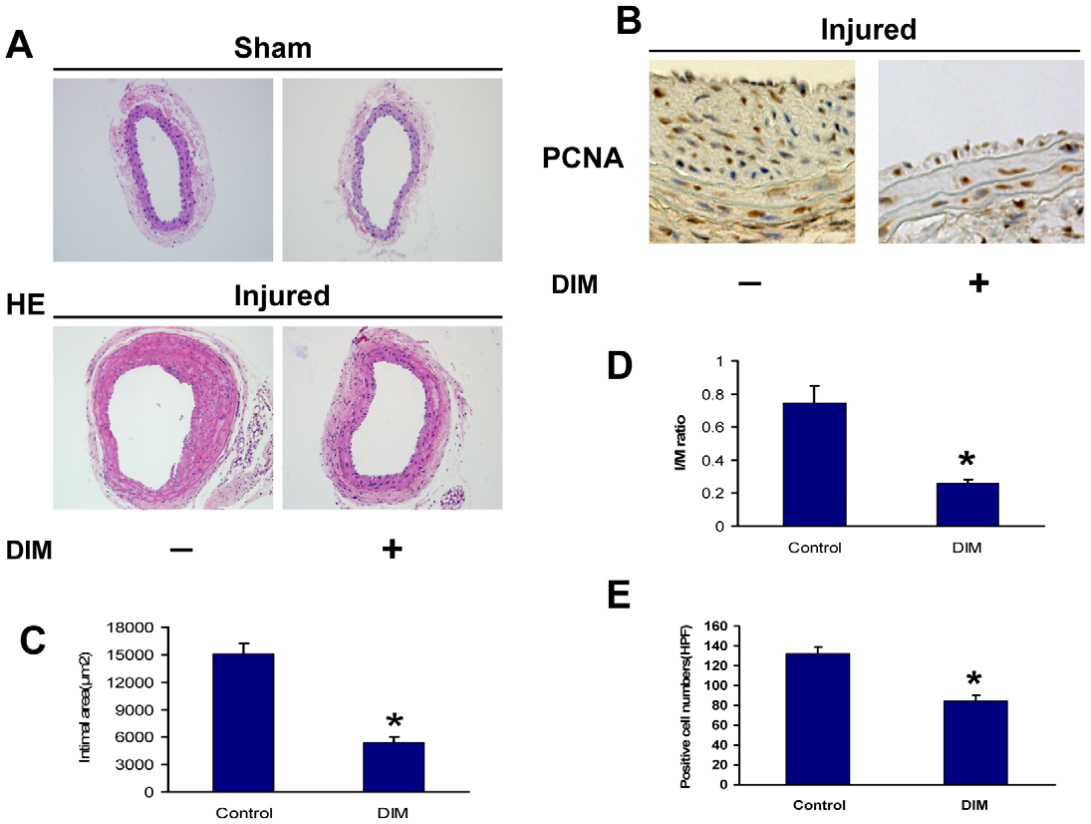
DIM prevents neointima formation induced by guidewire injury. A–B. Representative sections of the injured carotid artery of either an animal from the control group or the DIM-treated group are shown. C–D. Quantification of the intimal area and I/M ratios of carotid arteries of mice from either the control group or the DIM-treated group (n = 6, *P<0.01 versus injured control). E. Quantification of PCNA-positive cells of carotid arteries of mice from either the control group or the DIM-treated group (n = 9; *P<0.01 versus injured control).

To evaluate the effects of DIM on neointima formation, mouse carotid arteries were harvested 28 days after injury and subjected to morphometric analysis. Representative sections from control and DIM-treated injured carotid arteries are shown in Figure 7A. DIM inhibited neointima formation 28 days after guidewire injury to the carotid arteries (Figures 7A, C and D). The neointima area and I/M ratio in the DIM group (n = 6) were significantly reduced compared with the injured controls (n = 6) at 28 days after carotid injury (intimal area: 5,458.2±606 µm^2^ versus 15,036.7±1,220.7 µm^2^; I/M ratio: 0.26±0.02 versus 0.75±0.1). To assess VSMC growth, arterial sections were stained with anti-PCNA antibody and analyzed. DIM also significantly suppressed cell proliferation, as indicated by a reduced number of PCNA-positive cells 28 days after carotid injury (132.11±7.45 in controls, n = 9; 84±6.449 in the DIM group, n = 9) (Figure 7B and E).

### The effects of DIM on VSMC reendothelialization, apoptosis and inflammation in vivo

**Figure 8:**
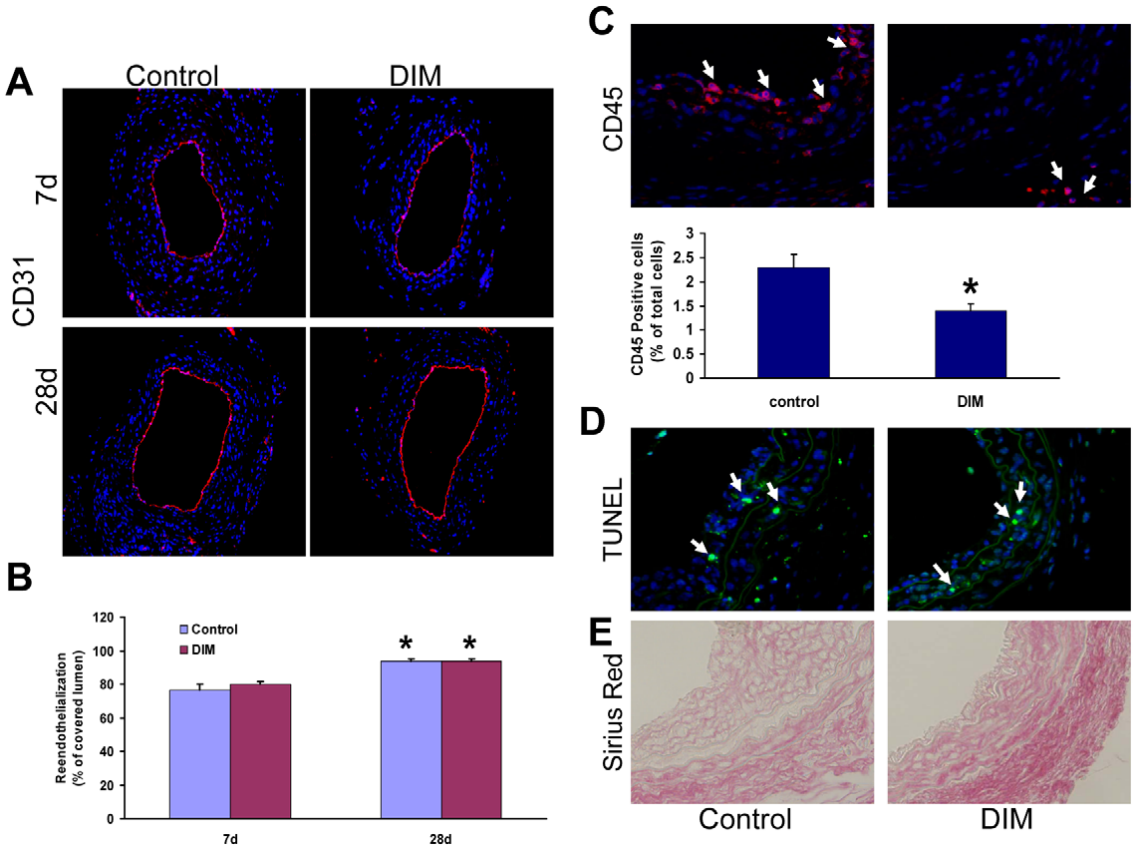
Effect of DIM on reendothelialization, inflammation, apoptosis, and extracellular matrix deposition in vivo. A. Representative immunohistochemical staining for CD31 at day 7 and day 28 after injury. B, Quantitative analysis showed no difference in the extent of reendothelialization between the 2 groups (n = 6, *P* = NS versus injured control, *P<0.05 versus day 7). C. Infiltration of inflammatory cells at 7days after vascular injury. Inflammatory cells were immunostained with anti-CD45 antibody. Arrows indicate positive cells (n = 6, *P<0.05 versus injured control). D. Apoptotic cells were assessed by TUNEL method at 7 days after injury. Arrows indicate TUNEL-positive cells. E. Sirius red staining of injured vessels (28 days). Collagen fibers were stained in red.

Given our data with HUVECs in vitro, we also investigated the effect of DIM on the reendothelialization of injured carotid arteries in vivo. Immunostaining of CD31 showed that the extent of reendothelialization was equivalent between control and DIM group at day 7 and day 28 after injury, confirming that administration of DIM did not impair reendothelialization (Figure 8A and B).

We also investigated whether DIM affected infiltration of inflammatory cells in injured vascular tissues. The number of CD45 positive cells in the injured vessels was significantly attenuated in DIM-administered mice (Figure 8C).

To evaluate the effect of DIM on cell apoptosis, TUNEL-positive cells were quantified. DIM did not augment apoptosis of VSMCs (Figure 8D). These results suggest minor contribution of apoptosis to the inhibition of neointima growth by DIM.

Sirius Red staining revealed that the collagen fibers were abundant in neointima and media tissues in both groups, there were no detectable differences in extracellular matrix (ECM) components between two groups (Figure 8E).

## Discussion

In this study, we characterized the biological effects of DIM on VSMCs in vitro and in vivo in injured mouse arteries. We demonstrated that DIM lessened PDGF-BB-induced VSMC phenotypic modulation and the development of neointima formation after vascular injury. We provided evidence that DIM stabilized p27^Kip1^ and downregulated cyclin D1. Subsequently, DIM prevented the proliferation and migration of VSMCs. These beneficial effects of DIM on VSMCs are associated with the inhibition of PDGF-Rβ phosphorylation and its downstream signaling pathways, including the MAPK, Akt/GSK-3β and STAT3 pathways. The efficiency of DIM was also observed in vivo because oral administration of DIM effectively prevented neointima formation in a murine model of wire-induced vascular injury. These results suggest that DIM might be a novel therapy for preventing injury-induced vascular remodeling.

It is well established that excessive VSMC proliferation and migration in the arterial walls plays a pivotal role in the development of neointima; therefore, inhibiting pathological VSMC proliferation and migration is a critical step in preventing and treating postangioplasty restenosis. Our results clearly demonstrated that DIM inhibited VSMC proliferation and PDGF-BB-induced DNA synthesis in a concentration-dependent manner. This antiproliferative effect of DIM was not due to cellular cytotoxicity, as demonstrated by the evans blue exclusion.

Cell proliferation is primarily controlled by regulation of the cell cycle. A growing body of evidence demonstrates that DIM causes cell cycle arrest in a variety of cancer cell lines; therefore, we hypothesized that DIM would block VSMC proliferation through cell cycle arrest. As we expected, our results showed that DIM treatment for 24 h led to G1 phase arrest, exhibited by a significant accumulation of cells in the G0/G1 phase and a reduction of cells in the S phase, which indicate that DIM inhibits cell cycle progression quite early in the G0/G1 phase. Modulation of the expression and function of cell cycle regulatory proteins provides an important mechanism for inhibiting cell growth. The D-type cyclins combine and activate CDK4 and CDK6 to phosphorylate and inactivate retinoblastoma (Rb) protein, which induces progression through the G1 phase of the cell cycle [21]. The activity of the cyclin/CDK complex depends on the balance of cyclins and CDK inhibitors (CKIs). One of the CKIs, p27^Kip1^, can bind to and inhibit a wide spectrum of cyclin/CDK complexes, including cyclin D-CDK4/6 and cyclin E-CDK2, and arrest cell growth at the G1 – G1/S boundary. Our experiment indicated that DIM treatment resulted in significant downregulation of cyclin D1-CDK4/6 and upregulation of p27^Kip1^, consistent with the inhibitory effect of DIM on VSMC proliferation. These observations suggest that the antiproliferative activity of DIM involves a multifaceted attack on multiple target molecules critically involved in growth inhibition. Over the last decade, several observations have shown a close link between cell cycle progression and cell migration. In addition to their involvement in cell cycle regulation, cyclin D1 and p27^Kip1^ have been reported as cell migration regulators. Previous studies have demonstrated that cyclin D1 promotes cell migration both in vitro and in vivo [23], [24], whereas reductions in cyclin D1 levels blocked PDGF-BB-induced migration. Cyclin D1 exerts its anti-migratory effect by inhibiting Rho-activated kinase II and thrombospondin 1 [23]. Unlike the consistent role of cyclin D1 in cell migration, the effects of p27^Kip1^ on cell migration are controversial. Besson et al. postulated that these differences may depend on cell type-specific variations in the relative balance between Rho and Rac activities and in the molecular interactions and subcellular localization of RhoA [25]. Results from different research groups have demonstrated that p27^Kip1^ inhibits cell migration in VSMCs [26], [27]. In the present study, DIM-induced upregulation of p27^Kip1^ and downregulation of cyclin D1 expression were consistent with DIM's inhibitory effect on VSMC migration. These results suggest that the effects of DIM on cyclin D1 and p27^Kip1^ expression may also involved in inhibiting VSMC migration. However, VSMC migration is a complicated process involving various signaling pathways and effector molecules; the exact role and mechanism of cell cycle protein in the process of inhibition of migration by DIM needs further investigation. On the whole, our study suggests that protection against neointima thickening by DIM might result from a combination of growth suppression and migration blockade.

Phenotypic modulation is an important phenomenon in VSMC activation. In response to vascular injury, the phenotype of VSMCs changes from quiescent, differentiated, and contractile to less differentiated and synthetic [28]. In addition to increased proliferation and migration, dedifferentiated VSMCs demonstrate decreased expression of smooth muscle-specific contractile markers, such as SM α-actin, SM22α and desmin [22]. This dedifferentiated phenotype contributes to the pathogenesis of restenosis after PCI. It is well established that PDGF-BB is a key mediator of VSMC phenotypic switching [29]. In accordance with previous studies, we observed that PDGF-BB decreased SM α-actin, SM22α and desmin expression, apart from enhanced VSMC proliferation and migration. More importantly, our in vitro study demonstrated that DIM treatment partly rescued the expression of SM α-actin, SM22α and desmin, accompanied by reduced cell proliferation and migration. These results suggest that DIM may halt the change toward a deleterious VSMC phenotype induced by PDGF-BB, which in turn contributes to the suppression of neointima formation.

Given that phenotypic modulation of VSMCs contributes to the formation of advanced atherosclerotic lesions and in-stent restenosis, DIM may be beneficial in these vascular injuries. In the present study, we used a well-established carotid injury model to investigate DIM's ability to protect against the neointima response to vascular injury. We demonstrated that DIM attenuated the increase in PCNA-positive cells in the neointima region and significantly reduced intimal hyperplasia upon injury to the mouse carotid artery. In addition to the antiproliferative effect, DIM was demonstrated to inhibit inflammatory cell recruitment, which may further contribute to the effective reduction of vascular lesion formation. These results bolstered our *in vitro* findings and provided direct evidence to support the notion that DIM could protect against pathological vascular remodeling after vascular injury. In our study, DIM was initiated after vascular injury and was able to prevent vascular remodeling; these results increase the clinical relevance of our findings. Furthermore, our data indicated that DIM neither induce apoptosis of VSMC nor exert toxic effect on VSMC. In addition, DIM did not show cytotoxic effect on HUVEC in vitro and did not affect reendothelialization in vivo. Therefore, due to its practicality and safety as a potential therapeutic application, DIM may represent an attractive molecule for treating vascular remodeling.

Although the entire mechanism of DIM-induced modulation of VSMC activity remains unclear, according to our study, DIM's effect on VSMCs is considered to be at least partly due to the inhibition of the Akt/GSK-3β, ERK1/2 and STAT3 signal pathways. Akt has various downstream targets, including GSK-3β. It is known that activated Akt phosphorylates GSK3β and decreases its catalytic activity [30]. The Akt/GSK3β pathway is implicated in multiple cellular processes, including migration and proliferation events downstream from growth factors, such as PDGF. We examined the activation of this signaling pathway. Consistent with previous studies, PDGF-BB induced rapid and sustained activation of Akt/GSK3β. More importantly, pretreatment with DIM facilitated the dephosphorylation of Akt and its substrate GSK3β, which meant the inactivation of Akt and reversed activation of GSK3β. GSK-3β activation has been found to induce cyclin D1 export from the nucleus to the cytoplasm for proteolysis, which decreases the availability of cyclin D1 [31]. Furthermore, GSK-3β could inhibit the translocation of the nuclear factor of activated T cells (NFAT) into the nucleus, which was recently found to induce cyclin D1 expression [24]. GSK-3β inhibition also has been shown to decrease expression of the CDK inhibitor p27^Kip1^ [32]. Moreover, Bianchi et al. have found that GSK-3β phosphorylates serine residues in focal adhesion kinase (FAK) to negatively modulate FAK catalytic activity, which is important in cell migration [33]. Based on these results, it is possible that the inactivation of Akt/GSK3β contributes to DIM's biological effects.

We also examined DIM's effect on the activation of ERK1/2 and STAT3, two other important players implicated in PDGF-BB-induced VSMC proliferation and migration. The ERK1/2 pathway has been found to participate in PDGF-induced downregulation of p27^Kip1^ and subsequent cell proliferation [34]. STAT3 can directly regulate cyclin D1 transcription [35]. Moreover, it appears that STAT3 activation is involved in VSMC inflammation and neointima formation [36], [37]. In a similar way, DIM also attenuated PDGF-stimulated phosphorylation of ERK1/2 and STAT3, this indicates that ERK1/2 and STAT3 are also potential targets of DIM.

The ability of DIM to inhibit PDGF-stimulated Akt, ERk and STAT3 phosphorylation suggests that DIM may mediate its effects on PDGF signaling by acting upstream of these molecules. PDGF binds to its cognate receptor, the PDGF-Rβ, leading to its phosphorylation on multiple tyrosine residues. This results in the recruitment and activation of specific signaling molecules, including ERK1/2, PI3K/Akt and STAT [38]. In the present study, we found a direct inhibitory effect of DIM on the phosphorylation of PDGF-Rβ. which might account for its profound in vivo effects. The mechanisms underlying the inhibition of PDGF-Rβ activity by DIM needs further investigation.

In summary, our data indicate that DIM inhibits VSMC phenotypic modulation induced by PDGF-BB and attenuates neointima formation in response to vascular injury. This process is associated with an inhibition in cyclin D1 expression and an increase in p27^Kip1^ accumulation via blockade of PDGF-Rβ phosphorylation and its downstream signal transduction. The present study suggest a potential of DIM as part of a therapeutic strategy for vascular proliferative disease, however, its safety especially during long term systemic application needs further evaluation.

## Supporting Information

Figure S1 Effect of DIM on viability of VSMCs and HUVECs. A. VSMCs were incubated in growth medium in the absence or presence of different concentrations of DIM for 48 h, and cell viability was evaluated by counting the number of cells that excluded the trypan blue dye (*P* = NS versus control group; n = 4). B, HUVECs were incubated in growth medium in the absence or presence of different concentrations of DIM for 48 h, and cell viability was evaluated by trypan blue exclusion (*P* = NS versus control group; n = 4).
(TIF)

Figure S2 Inhibitory effects of DIM on PDGF-Rβ, Akt, GSK-3β, ERK1/2, and STAT3 activation in PDGF-BB-stimulated VSMCs. Bar graphs showing the quantification of the Western blots; results are expressed as percentages of the control (*P<0.05 versus PDGF alone, n = 3).
(TIF)
