# Supplementary figures and images for: 3,3′Diindolylmethane Suppresses Vascular Smooth Muscle Cell Phenotypic Modulation and Inhibits Neointima Formation after Carotid Injury
Source: PLoS One. 2012 Apr 10;7(4):e34957. doi: 10.1371/journal.pone.0034957 (PMC3323601; doi:10.1371/journal.pone.0034957)

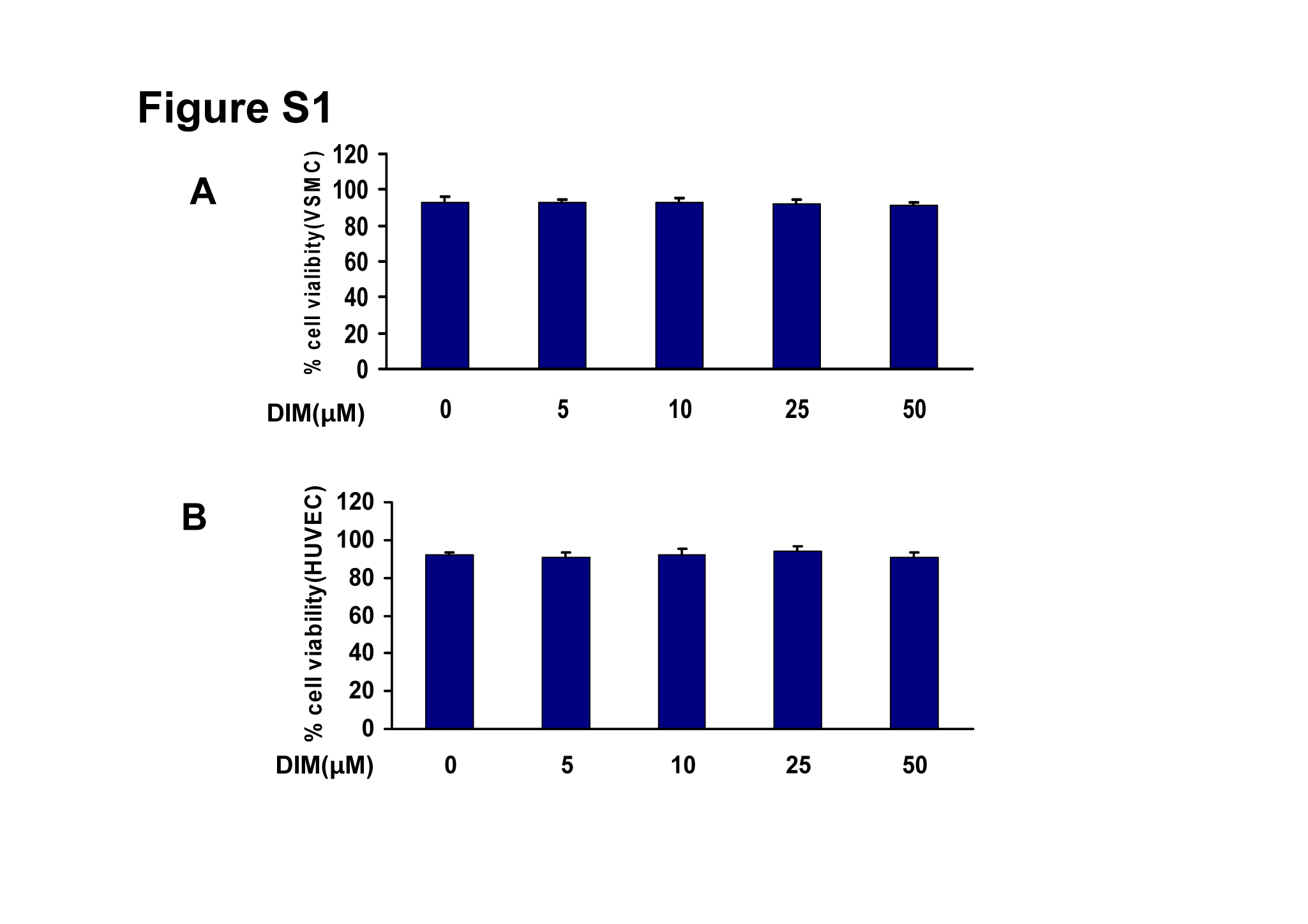

Supplement: Figure S1 — Effect of DIM on viability of VSMCs and HUVECs. A. VSMCs were incubated in growth medium in the absence or presence of different concentrations of DIM for 48 h, and cell viability was evaluated by counting the number of cells that excluded the trypan blue dye (P = NS versus control group; n = 4). B, HUVECs were incubated in growth medium in the absence or presence of different concentrations of DIM for 48 h, and cell viability was evaluated by trypan blue exclusion (P = NS versus control group; n = 4). (TIF) [file pone.0034957.s001.tif]

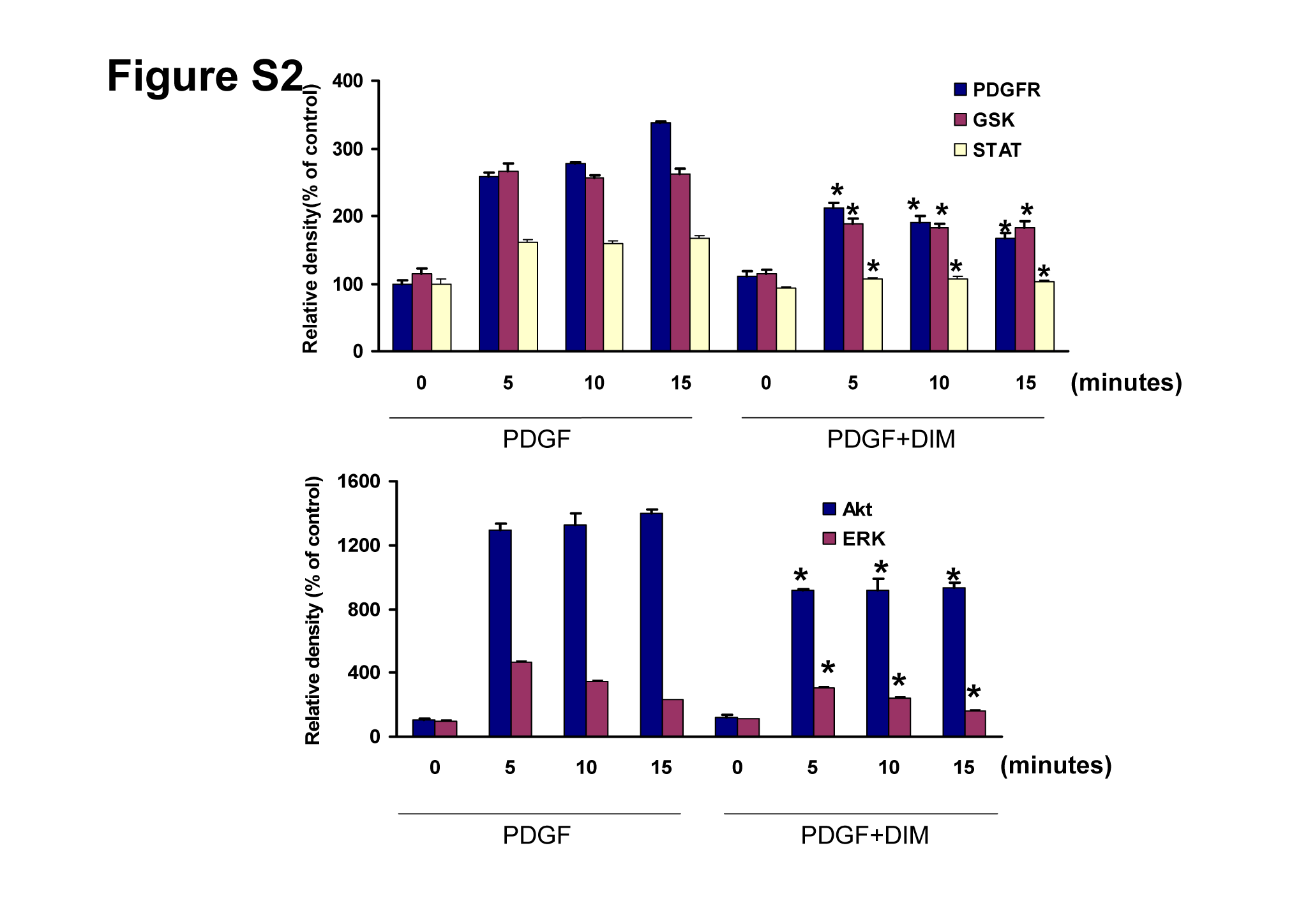

Supplement: Figure S2 — Inhibitory effects of DIM on PDGF-Rβ, Akt, GSK-3β, ERK1/2, and STAT3 activation in PDGF-BB-stimulated VSMCs. Bar graphs showing the quantification of the Western blots; results are expressed as percentages of the control (*P<0.05 versus PDGF alone, n = 3). (TIF) [file pone.0034957.s002.tif]
